# Autistic trait interactions underlie sex-dependent facial recognition abilities in the normal population

**DOI:** 10.3389/fpsyg.2013.00286

**Published:** 2013-05-31

**Authors:** Jeffrey M. Valla, Jeffrey W. Maendel, Barbara L. Ganzel, Andrew R. Barsky, Matthew K. Belmonte

**Affiliations:** ^1^Department of Human Development, Cornell UniversityIthaca, NY, USA; ^2^West Virginia School of Osteopathic MedicineLewisburg, WV, USA; ^3^Perelman School of Medicine, University of PennsylvaniaPhiladelphia, PA, USA; ^4^Department of Psychology, Nottingham-Trent UniversityNottingham, UK; ^5^The Groden CenterProvidence, RI, USA

**Keywords:** autism, cognitive variation, sex differences, face recognition, face processing

## Abstract

Autistic face processing difficulties are either uniquely social or due to a piecemeal cognitive “style.” Co-morbidity of social deficits and piecemeal cognition in autism makes teasing apart these accounts difficult. These traits vary normally, and are more separable in the general population, suggesting another way to compare accounts. Participants completed the Autism Quotient survey of autistic traits, and one of three face recognition tests: full-face, eyes-only, or mouth-only. Social traits predicted performance in the full-face condition in both sexes. Eyes-only males' performance was predicted by a social × cognitive trait interaction: attention to detail boosted face recognition in males with few social traits, but hindered performance in those reporting many social traits. This suggests social/non-social Autism Spectrum Conditions (ASC) trait interactions at the behavioral level. In the presence of few ASC-like difficulties in social reciprocity, an ASC-like attention to detail may confer advantages on typical males' face recognition skills. On the other hand, when attention to detail co-occurs with difficulties in social reciprocity, a detailed focus may exacerbate such already present social difficulties, as is thought to occur in autism.

## Introduction

Autism Spectrum Conditions (ASC) are defined by social-communicative deficits and behavioral inflexibility (American Psychiatric Association, 1994). Two common social deficits are face processing difficulties (Dawson et al., 2005), and eye gaze aversion (Klin et al., 2002). When viewing a video clip of a social scenario, for instance, individuals with autism spend less than half as much fixation time on characters' eyes than do individuals without autism, and about twice as much time looking at mouths and bodies (Klin et al., 2002).

To explain this social deficit, some suggest ASC individuals are less *interested* social stimuli (Klin et al., 2002), because social stimuli are, at a neural level, less rewarding for them. Social inattention may, in turn, negatively affect the development of neural networks subserving face processing (Dawson et al., 2005; Sterling et al., 2008). An alternative to this social-first model is a cognitive/perceptual model: impaired face processing may stem from a domain-general piecemeal perceptual style (Dawson et al., 2005, p. 403). ASC individuals may process faces by attending to shape and peripheral details, rather than central features (Hadjikhani et al., 2004). Although distinguishing between these models is a starting point for understanding origins of face processing deficits, this distinction need not imply mutual exclusivity. Inattention to social cues may draw one's attention to the physical configurations and individual components of faces; likewise, a piecemeal processing style may render meaningless the emergent, social properties arising from processing facial components in concert.

Evidence from fMRI studies supports both models. Supporting the social-first model, when viewing and processing faces, ASC individuals hypoactivate the fusiform gyrus, a brain region physiologically associated with face processing (Dalton et al., 2005). The same study also found that fixation time on the eyes is in ASC individuals is positively and strongly associated with hyperactivation of the amygdala, irrespective of facial emotion, gaze orientation, or facial familiarity (Dalton et al., 2005). This suggests stress reactivity that is specific to social stimuli such as eye gaze. It also suggests that social stimuli may be less rewarding to autistic individuals in the *negative* sense, inducing fear responses that autistic individuals learn to avoid by actively avoiding social stimuli. (Of course, active avoidance and disinterest models could be mutually reinforcing, rather than mutually exclusive, accounts of autistic individuals' lack of social engagement). Supporting the cognitive/perceptual model, viewing faces activates the inferior temporal gyrus in ASC individuals, a region activated when non-autistic individuals view objects (Sterling et al., 2008).

### Present study

Recent evidence indicates that autism represents an extremity on a spectrum of cognitive and social variation extending throughout the normative population (Constantino and Todd, 2003). As others have recognized, teasing apart social and non-social models of ASC may be done most effectively by making use of the entire population variance (Happé et al., 2006), as social and non-social ASC traits are likely more separable in the normative range. The present study used a sample of typically developing adults to examine whether individual variation in ASC social interaction deficits and/or detail-oriented cognitive “style” predict individual differences in face recognition ability.

Participants completed the Autism Spectrum Quotient (AQ) as a measure of autistic social deficits and preference for details. Face recognition was assessed using the Benton face recognition test (Benton et al., 1983); to test for a normative analog of the autistic tendency to avoid the eye region in favor of mouths, subjects completed one of three versions: the standard version (Full Face), a version with only eye region visible (Eyes-Only), or only the mouth region visible (Mouth-Only), and performances across conditions were compared.

If stress reactivity during eye contact drives normative differences in face processing, as in ASC, self-reported ASC-like social deficits—such as those inventoried by the AQ—should better predict face recognition ability than a self-reported preference for details (also measured by the AQ). Those with fewer ASC-like social deficits should have the advantage in the Full Face and Eyes-Only test versions, and those with more ASC-like social deficits should have the advantage in the Mouth-Only version. If, on the other hand, piecemeal processing underlies ASC face processing deficits, the configural approach used by those reporting low preference for details would have the advantage in the Full-Face version, whereas in both the Eyes-Only and Mouth-Only versions, a preference for details would be advantageous. Finally, based on findings that males, but not females, may apply detail-oriented strategies to face processing (Valla et al., 2010), we hypothesized a sex-dependent pattern in which a preference for details and face processing would be more closely related in males than females. Specifically, we hypothesized that a preference for details would be more closely linked to Benton performance in males than females across our three versions of the Benton test.

Whilst we have here posed social and cognitive models as two alternative hypotheses to be teased apart in the present study, it is important to reiterate that these two possibilities need not be mutually exclusive. On the contrary, autistic social and cognitive functioning may better understood as a function of interactions between social and non-social traits, than as the additive effect of co-present social and non-social traits. Use of a neurotypical sample is important in this respect because the greater separability of social and non-social autism-like traits in the typically developing population allows for teasing apart the effects of social and non-social traits; and allows for comparisons between the strength of their additive effect vs. any interactive, synergistic effects emerging from the co-presence of such traits.

## Materials and methods

94 (46 females, 48 males) undergraduate students (*M* = 20.7 years; *SD* = 2.4 years) were recruited from a large Northeastern U. S. University as part of a larger study, approved by the university's Institutional Review Board. Prospective participants were screened to ensure none had received ASC diagnosis, or had any first degree relative with ASC diagnosis. Participants completed the AQ—a self-report survey of subclinical ASC social, cognitive, and behavioral traits (Baron-Cohen et al., 2001b)—and one of three versions of the Benton face recognition test: the standard Benton face recognition test with the full face visible (Full Face, *n* = 32), a modified test with only eye regions visible (Eyes-Only, *n* = 30), and a test with only mouths visible (Mouth-Only, *n* = 32).

The AQ was scored as in Austin's (2005) four-point Likert scale (1–4), except a symmetric scale of −2, −1, +1, +2 was used. In addition to the full-scale AQ score, the five AQ subdomain scores validated by Austin (2005), attention switching (AQAttSw), communication (AQComm), details/patterns orientation (AQDet), imagination (AQImag), and social skills (AQSS) were computed. Valla et al.'s (2010) factor analysis of the five AQ subdomains was replicated, indicating two underlying factors; the first included AQAttSw, AQComm, AQImag, and AQSS subdomains which together comprised a *Social Interaction* (AQSocInt) category; the second factor included the AQDet subdomain. Based on this, composite AQSocInt scores were calculated by summing AQAttSw, AQComm, AQImag, and AQSS subscores.

An analysis of variance and a multiple regression model evaluated performance on the Benton face recognition test (dependent variable) as a function of test condition (Full Face, Eyes-Only, Mouth-Only), Sex, AQSocInt score, and AQDet score. Prior to analysis, the two continuous independent variables (AQSocInt, AQDet) were centered and standardized so as to reduce the possibility of multicollinearity and to increase interpretability across measures in our moderational models (Dawson and Richter, 2006). A summary of condition, numbers of subjects per group, means, and standard deviations of test scores, both by sex and in total, is listed in Table 1.

**Table 1:** **Summary of means and standard deviations, by Benton Test condition and sex**.

**Benton condition**	**Sex**		**Benton score**	**AQSocInt**	**AQDet**
Eyes-only	F	*N*	14	14	14
		*M*	43.57	−30.71	1.64
		*SD*	2.10	19.97	6.03
	M	*N*	16	16	16
		*M*	44.06	−25.50	−1.38
		*SD*	2.05	20.10	8.17
Mouth-only	F	*N*	18	18	18
		*M*	40.78	−28.44	0.83
		*SD*	3.75	17.42	5.86
	M	*N*	14	14	14
		*M*	39.14	−24.21	2.29
		*SD*	2.03	14.86	4.83
Full face	F	*N*	14	14	14
		*M*	47.71	−30.50	2.00
		*SD*	3.85	19.86	6.52
	M	*N*	18	18	18
		*M*	46.50	−20.94	−0.17
		*SD*	2.64	18.57	5.96

AQSocInt, AQ Social Interaction Composite Subscore; AQDet, AQ Details/Patterns Subscore.

## Results

A 2 (Sex) × 3 (Benton condition) analysis of variance revealed a significant main effect for Benton condition, *F*_(2, 88)_ = 48.95, *p* < 0.001, but no Sex × Benton condition interaction and no main effect of Sex. *Post-hoc*, Bonferroni-corrected comparisons between Benton conditions revealed significant differences between all three conditions, with the Full-Face group outperforming the Eyes-Only group, and both these groups outperforming the Mouth-Only group (all *p*'s < 0.001).

**Figure 1:**
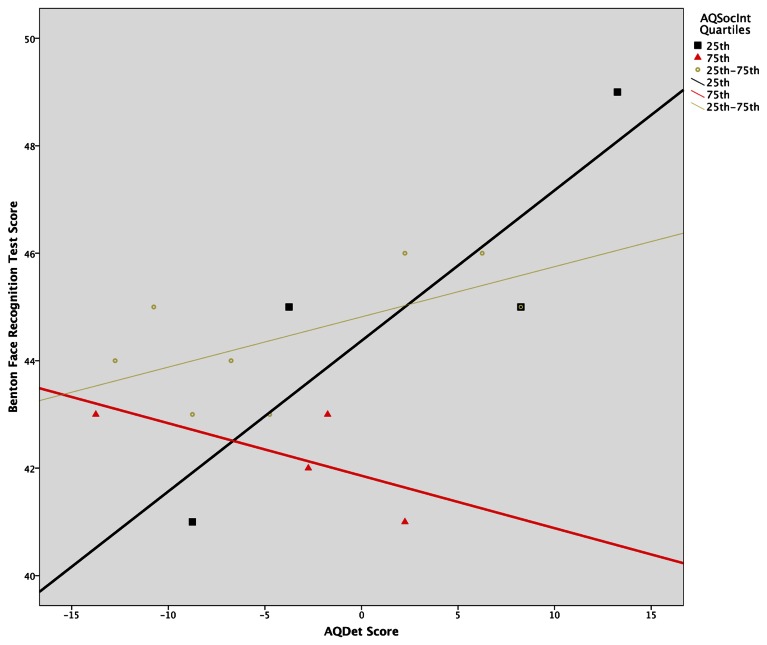
**AQ Details/Patterns score(AQDet) × Benton test score in Male Eyes-Only group, by AQ social interaction score (AQSocInt) Quartile.** Splitting the 46 female and 48 male subjects across the three Benton testing conditions yielded subgroups of 16 or 15 subjects per condition, resulting in four or in two cases 3 subjects per AQSocInt score quartile. Significance of the contrasting plots within the above Figure should, however, be interpreted in the context of the full number of 16 or 15 individuals per condition, as quartile distinctions were used solely for graphing purposes (i.e., to bring interaction effects into greater relief in the above Figure). Quantitative significance levels (tail probabilities) are provided in the text.

A multiple regression model using generalized linear regression revealed a significant four-way interaction between all predictors (*Wald c*^2^ = 8.54, *df* = 2, *p* = 0.014). To probe this interaction, separate linear models were run for each of the six Sex × Benton condition subgroups. This analysis revealed a significant AQSocInt × AQDet interaction in the Male, Eyes-Only subgroup, *F*_(1, 12)_ = 6.88, *p* = 0.022, as well as a significant main effect for AQSocInt in both the Male, *F*_(1, 16)_ = 7.62, *p* = 0.014, and Female, *F*_(1, 12)_ = 9.98, *p* = 0.008, Full Face groups. All three effects remained significant after correcting for multiple comparisons, via comparison of *p*-values to Benjamini-Hochberg derived *q*-values corresponding to the rank order of each *p*-value (Benjamini and Hochberg, 1995). These three effects were then plotted to ascertain their directions; a plot of the AQSocInt × AQDet interaction in the Male, Eyes-Only subgroup, using AQSocInt quartile split groups to demonstrate the interaction, is provided in Figure 1.

In the Male, Eyes-Only subgroup, AQDet scores were more positively associated with Benton performance when AQSocInt scores were low than when they were high. For those with the highest AQSocInt scores, the association between AQDet and Benton score actually became negative: that is, in individuals reporting the most autistic social impairments, attention to detail predicted deficits in face recognition; at this extreme of normal population variation, autistic social, and non-social traits began to synergise. Although one potentially influential outlier was observed, the significant interaction remained when the analyses were repeated excluding this potential outlier. In both Male and Female Full Face subgroups, the association between AQSocInt and Benton test scores was negative, with higher AQSocInt scores being associated with lower Benton test performance.

## Discussion

The current study revealed face processing synergies between social and non-social ASC traits in typically developing individuals. First, when face recognition was tested using whole faces, the presence of self-reported ASC social deficits was predictive of poorer performance on the Benton face recognition test in both sexes; a preference for details was, on the other hand, unrelated to face recognition in this condition, in both sexes. More interesting, though, is the sex-dependent result in the Eyes-Only Benton test condition. We expected the Eyes-Only test condition to amplify the association between social deficits and face recognition found in both sexes in the Full Face condition. Instead, males in the Eyes-Only condition exhibited an interaction between ASC social deficits and detail-oriented processing biases that significantly predicted eyes-based face recognition ability. In men reporting few social deficits, a preference for details was positively associated with face recognition; whereas in men reporting many social deficits, the association between a preference for details and face recognition became negative. Meanwhile, neither social nor non-social ASC traits predicted females' face recognition ability in the Eyes-Only condition.

One interpretation of the male pattern is that males' face processing strategy changed when their focus was drawn to the eye region. Although males' face recognition ability was related to social deficits, as in the Full Face condition, males in the Eyes-Only condition may have also engaged detail-oriented processing to solve the inherently social problem of face recognition, assuming that a self-reported preference for details translates into preferences of cognitive strategy. For those reporting few social deficits, this alternative strategy appeared to aid face recognition, as a preference for details was more positively associated with face recognition performance when few social deficits were reported. This strategy presumably allows these individuals to recognize faces by “disembedding” relevant features from the eye region, like a social analog of the EFT, and matching them to the eye regions of matching faces. However, the more social deficits reported, the more negative the association between a preference for details, and face recognition performance, suggesting the alternative, piecemeal strategy may synergize with social deficits, rendering face recognition even harder than in the case of social deficits alone.

These two interpretations—that of a detail-oriented approach to social problem solving in males reporting fewer social deficits, and that of a social deficit-preference for details synergy that leads to greater social deficits in males reporting more social deficits—may represent two sides of a single phenomenon. The former is akin to Golan and Baron-Cohen's (2006) concept of ‘systemizing empathy’ or applying detail-oriented cognitive/perceptual approaches to social contexts. For instance, ASC individuals can exhibit excellent facial emotion recognition when piecemeal-oriented tests are used (Evers et al., 2011). Similar patterns have been found in typically developing males. Decreases in males' performance on the “Reading the Mind in the Eyes” test (Baron-Cohen et al., 2001a) has been associated with fields of study requiring attention to detail (e.g., mathematics, computer science) yet, in these males, EFT scores and AQDet subscores, were both *positively* related to these same facial emotion skills (Valla et al., 2010). In another study, self-reported attention to detail was positively related to facial emotion reading ability, in male participants (Voracek and Dressler, 2006).

That preference for details was related to face recognition in the Eyes-Only but not the Full Face condition in the current study may mean that in most typical individuals such strategies are not automatically engaged when focus is placed on all components of the face at once (i.e., Full Face condition), an inherently more global stimulus than the eye region. This would reflect a subclinical case of social and non-social ASC traits presenting as though they stem from a common syndrome, yet admit dissociable neurological origins (Happé et al., 2006). If, for instance, a detail-oriented processing bias in the presence of social deficits heightens these deficits—by bringing a hyper-focus to eye regions that are already aversive for those with autistic social traits—then aversion to social stimuli may become amplified during the course of development. In this way, the extent of social dysfunction in autism may reflect emergent social deficits arising from a synergy of ASC social and non-social traits.

If detail-oriented processing is engaged when males' focus is drawn to facial subcomponents such as the eyes, then the fact that preference for details was unrelated to face recognition in the Mouth-Only condition appears counterintuitive. On the other hand, the ASC social deficits that were related to face recognition in the Full Face condition in both sexes and the Eyes-Only condition in males were also unrelated to performance in the Mouth-Only condition; and across both sexes those in the Mouth-Only condition performed significantly worse than the two other conditions. So mouths may be lacking useful face recognition information, producing a floor effect in which participants reporting few social deficits had as much difficulty as those reporting many social deficits.

Why would “systemizing empathy” be male-specific? Previous research on normative variation in ASC traits indicates that social deficits and a detail-oriented cognition are more inversely related in males than females, in whom they are more orthogonal (Voracek and Dressler, 2006; Valla et al., 2010); in males, a detail-oriented style comes with social tradeoffs. Males with a preference for details thus may use an alternative, compensatory, piecemeal processing strategy to compensate for the social difficulties that come with a detail-oriented style, similar to what others have found with ASC samples (Evers et al., 2011). Sex-dependent social/non-social co-variance is not unique to research on normative variation in ASC traits. Cognitive sex difference theorists have recently cited sex-dependent co-variance between social-communicative and spatial/numerical skills in alternative hypothesis for the underrepresentation of women in science and engineering fields: being more likely than their male counterparts to be skilled in both social and non-social realms provides math-savvy women with more career options (Ceci and Williams, 2011; Valla and Ceci, 2011); math-savvy men, by contrast, experience social-communicative tradeoffs with these skills, limiting their choices to math-intensive fields.

In terms of the broader debate over social-first vs. non-social models of ASC, the current study supports an alternative, developmental synergy account. Although the interaction between social and non-social skills in males in the Eyes-Only condition demonstrates the influence that piecemeal processing can have on face processing, whether piecemeal processing aids or hinders face processing depends on the level of social deficits. This suggests that dynamic interactions between ASC social and non-social traits take place at the *behavioral* level; in the context of face processing, for instance, social/non-social interactions may determine face recognition strategy. The presence of such interactions would lend support to recent Interactive Specialization models of autistic and normative development, providing a mechanism by which initially independent social and non-social ASC traits could become inextricably linked during development (Valla and Belmonte, in press).

Importantly, this interpretation is based on correlational findings; further investigation is required to confirm such causal directionality. If the hypothesized causal chain cannot be confirmed, another possibility would be a singular mechanism of autism with incomplete penetrance: that is, a single cause that doesn't manifest overtly unless its effects exceed some homeostatic threshold (a threshold that could arise even beyond the limit of the autism spectrum, with consequences becoming more severe and clinical as the degree of violation of this threshold increases). Such a mechanism might be acting to make face recognition more “autistic” (i.e., lesser) in the same individuals in whom it also renders attention to detail more “autistic” (i.e., greater) and social competence more “autistic” (i.e., lesser).

Aside from its correlational nature, the primary methodological weaknesses of this study are the modest sample size, and the lack of within-subjects design. As with the correlational design, the cell sizes of the four-way interaction that arose mean the conclusions drawn from probing this interaction should be considered with some caution. A within-subjects designs (i.e., each subject exposed to all three facial stimuli conditions) would have allowed us to test the assumption of stable face processing “style,” by seeing if individuals' performance can be manipulated by providing more or less facial information. This design was not used for the present study because dividing the Benton test items into different tests (full face, mouth-only, eyes-only) would greatly reduce the amount of individual variance possible in each condition/test, a crucial aspect of this individual difference investigation.

Thus, future investigations can improve upon the present, exploratory study in three ways. First, by using an experimental design (e.g., facial stimuli varying in degree of fragmentation, such that “piecemeal” processing is favored in some conditions) to test the hypothesized causal directionality more directly than the present correlational study. Second, by using sample sizes allowing for a clearer interpretations of the four-way interactions that arose in this initial investigation. Finally, future studies can improve upon the present work by using a within-subjects design, and a face recognition test that preserves the degree of individual variation afforded by the Benton; the addition and validation of new Benton items to may be the best way to accomplish the latter.

In addition to these methodological limitations, the main theoretical limitation of the present study is the use of neurotypical sample for testing hypotheses concerning autism. Even if autism does represent an extremity on a continuum that is contiguous with neurotypical human cognitive variation, the unique constellation of atypical behaviors that constitute clinical extremity implies emergent properties of extremity that cannot be predicted from neurotypical patterns. As discussed in the introduction, however, the use of neurotypical subjects is also a strength of this study; greater separability of social and cognitive “autistic” traits in the neurotypical population makes it easier to tease apart and explore interactions between traits that are by definition co-morbid in clinical cases.

## Conclusion

Beyond the autism spectrum, individual differences in ASC social and non-social traits predict face recognition ability; but the nature of these predictions depends on sex and the type of facial information provided. Participants of both sexes reporting more social deficits were disadvantaged at full face recognition, indicating a uniquely social processing approach. Limiting face recognition to the eye region may alter some males' face processing strategies, however, toward a piecemeal approach. The effectiveness of this strategy depends on the presence of social deficits: in socially competent individuals, a piecemeal strategy works to their advantage, whereas in those reporting many social deficits it is a disadvantage to face recognition. This socially-dependent effect of a non-social cognitive trait may help explain how non-social traits like detail-oriented processing exacerbate social deficits in autism.

At the broadest level, this study further supports the idea that exploring interactions between ASC cognitive and social traits in the normative range can illuminate these processes in ways that clinical samples cannot. This study also demonstrates for the first time that the continuity between ASC and typical development applies to the domain of face recognition.

### Conflict of interest statement

The authors declare that the research was conducted in the absence of any commercial or financial relationships that could be construed as a potential conflict of interest.
